# Effects of moxibustion for constipation treatment: a systematic review of randomized controlled trials

**DOI:** 10.1186/1749-8546-5-28

**Published:** 2010-08-05

**Authors:** Myeong Soo Lee, Tae-Young Choi, Ji-Eun Park, Edzard Ernst

**Affiliations:** 1 Division of Standard Research, Korea Institute of Oriental Medicine, Daejeon 305-811, South Korea; 2 Complementary Medicine, Universities of Exeter & Plymouth, Exeter, EX2 4NT, UK

## Abstract

Several studies reported that moxibustion was effective in treating constipation. This systematic review assesses the clinical evidence for or against moxibustion for treating constipation. Twelve databases were searched from their inception to March 2010. Only randomized clinical trials (RCTs) were included if they compared moxibustion with placebo, sham treatment, drug therapy or no treatment. The methodological quality of these RCTs was assessed with the Cochrane risk of bias analysis. All three RCTs included in the study had a high risk of bias. Two included studies found favorable effects of moxibustion. The third RCT showed significant effects in the moxibustion group. Given that the methodological quality of all RCTs was poor, the results from the present review are insufficient to suggest that moxibustion is an effective treatment for constipation. More rigorous studies are warranted.

## Background

Chronic constipation is a prevalent health condition with patients typically having bowel movements twice a week or less for at least two consecutive weeks or longer. The Rome II criteria define chronic constipation on the basis of two or more of the following symptoms at least 25% of the time for at least 12 weeks in the preceding year: straining at defection, lumpy/hard stools, sensations of incomplete evacuation and three or fewer bowel movements per week [1]. Currently, there is no optimal therapeutic solution for this condition.

Acupuncture and moxibustion are increasingly used for the treatment of gastrointestinal (GI) diseases [2-4]. Moxibustion is a Chinese medicine treatment whereby an acupoint is stimulated by the heat generated from burning *Artemisia vulgaris *[5]. Direct moxibustion is applied to the skin surface, whereas indirect moxibustion is performed with some insulating materials (e.g. ginger, salt) placed between the moxa cone and the skin [5]. The heat is then used to warm the skin at the acupoint.

Chinese medicine has a unique approach to diagnosis of constipation [6]. According to Chinese medicine theory, there are four constipation patterns, namely differentiation constipation (including heat constipation), cold constipation, *qi *constipation and deficiency constipation. The draining method employing filiform needles is used to treat heat constipation and *qi *constipation [7]. In general, moxibustion is used to treat cold constipation, and deficiency constipation [8].

A possible explanation is that the heat stimulates acupoints thereby increasing *qi *circulation and relieving *qi *stagnation [9], leading to increased frequency of bowel movement.

Among three available systematic reviews on acupuncture for constipation [10-12], two reviews regarded constipation as part of a range of GI disorders [11,12] and included only one uncontrolled observational study. The third systematic review focused on auriculotherapy [10] and included only non-randomized clinical trials. A Cochrane protocol is also available [13].

The present review aims to summarize and evaluate the evidence from randomized controlled trials (RCTs) that examined the effectiveness of moxibustion as a treatment for constipation.

## Methods

### Data sources

MEDLINE, AMED, EMBASE, CINHAL, five Korean Medical Databases (i.e. Korean Studies Information, DBPIA, the Korea Institute of Science and Technology Information, KoreaMed and the Research Information Service System), China National Knowledge Infrastructure (CNKI), Cochrane Library (2010, Issue 2) and Japanese electronic database (Japan Science and Technology Information Aggregator, Electronic-J-STAGE) were searched from their inceptions to March 2010: Search terms used were 'moxibustion' AND 'constipation or obstipation or costiveness' in Korean, Chinese or English. Relevant journals (i.e. *Focus on Alternative and Complementary Therapies *and *Forschende Komplementarmedizin*) were electronically searched up to March 2010. Moreover, references of all obtained articles were searched. Our own files were manually searched. Hard copies of all potentially relevant articles were obtained and read in full.

### Study selection

Inclusion criteria were (1) RCTs involving human patients with any type of constipation [e.g. primary (functional) constipation and secondary constipation (complication from other conditions)] treated with moxibustion; cause of constipation was not considered; (2) placebo controlled or controlled trials against a conventional treatment (e.g. drug therapy or another active treatment) or against no treatment; (3) dissertations and abstracts with substantial contents. Exclusion criteria were (1) trials of moxibustion coupled with other therapies; (2) trials for 'warm acupuncture' (i.e. moxibustion on top of an acupuncture needle).

### Data extraction, quality and validity assessment

Two reviewers (TYC, JEP) independently read all articles and extracted data from the articles according to predefined criteria (Table 1). Risk of bias was assessed with the four criteria of Cochrane classification, namely sequence generation, incomplete outcome measures, blinding and allocation concealment [14]. As it is virtually impossible to blind the moxibustion therapists from the treatment, we evaluated patient and assessor blinding separately. Disagreements were resolved by discussion between the two reviewers (TYC, JEP). A third reviewer (MSL) was consulted if necessary. There was no disagreement between the two reviewers on the risk of bias.

**Table 1:** Summary of randomized clinical studies of moxibustion for constipation

First author	Sample size Condition Age range or mean age (years) Gender (M/F) Diagnosis criteria Chinese Medicine Diagnosis	Intervention group (regimens)	Control group (regimens)	Main outcomes	Results (*P *value, RR, 95%CI)	Adverse events
Du (2008) [15]	160 postpartum women 23-42, (0/160) n.r. Rome II (Once per 10 days) n.r.	Moxa (once daily, total 6 treatments, n = 80) Tongbian acupoint (Bilateral) Indirect	Drug (Glycerine Enema, once daily for 14 days, total 14 treatment, n = 80)	Response rate*	*P *< 0.01, RR 1.27, 95%CI [1.13, 1.42]	n.r.
Li (2001) [16]	60 n.r. Moxa: 51, (12/28) Drug: n.r. (similar with moxa group) n.r. Gastrointestinal heat accumulation, body fluid deficiency	Moxa(once daily, total 5 treatment, n = 40) CV8 Indirect	Drug (Glycerine Enema, once daily for 5 days, n = 20)	Response rate^†^	*P *< 0.01, RR 1.50, 95%CI [1.08,2.08]	n.r.
Kwon (2005) [17]	36 stroke patients n.r. (20/16) Rome II (Twice weekly) None	Moxa (total 28 treatment for 4 weeks, n = 17) ST25(Bilateral) Indirect	No treatment (n = 19)	1) Stool frequency 2) Constipation Assessment Scale	1) *P *= 0.0001 2) *P *= 0.0001	Itching, skin eruption, eyes stinging from the smoke

CAS: Constipation Assessment Scale, n.r: not reported; CVD: cardiovascular disorders; * 1) Recovery: 1-2/d bowel movement, discharge unobstructed, without the help of laxatives; 2) Improvement: defecation shorter time than before treatment, alleviate symptoms, but the need to laxative; 3) Ineffective: general and local symptoms did not improve; ^† ^1) Markedly effective: fecal excretion of smooth, no pain,1~2 time/d; 2) Effective: constipation improved, excretion 1 time/d; 3) Ineffective: after a course of treatment after treatment, no obvious improvement in constipation symptoms.

### Outcome measures and data synthesis

All clinical endpoints including stool frequency per week and Constipation Assessment Scale (CAS) were considered with the main outcome measure being the response rate from patients with constipation. We did not evaluate the outcomes related to surrogate endpoints. The differences between the intervention and control groups were assessed. Relative risk (RR) and 95% confidence intervals (CIs) were calculated for each study with Cochrane Collaboration's Review Manager (RevMan) software (Version 5.0 for Windows, Nordic Cochrane Center, Denmark). We considered a *P *value less than 0.050 to be statistically significant. Summary estimates of the treatment effects were calculated using the more conservative approach of a random effects model. Differences compared with a placebo control were considered relevant in the context of this study. Statistical heterogeneity was evaluated using a χ^2 ^test and I^2 ^statistics (low = 25%; moderate = 50%; high = 75%). In the case of heterogeneity, we attempted to identify and explain the heterogeneity using subgroup analysis. Subgroup analysis was performed for subsets of studies. Where more than ten studies were available, we assessed publication bias using a funnel plot or Egger's regression test. Post hoc sensitivity analyses were performed to test the robustness of the overall effect.

## Results

### Study characteristics

**Figure 1:**
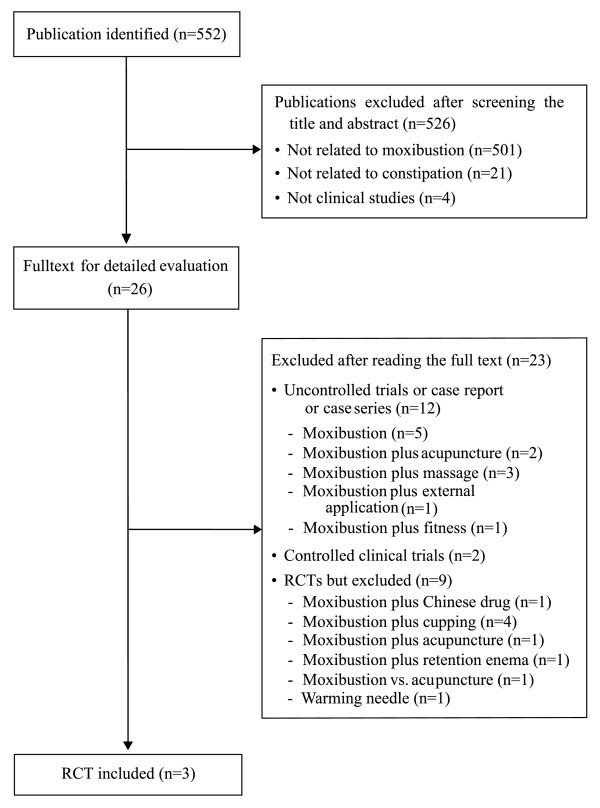
**Flowchart of trial selection process**. RCT: randomized clinical trial

Our searches identified 552 potentially relevant studies. Of these articles, 549 studies were excluded for reasons outlined in Figure 1. Table 1 lists the key data from the three included RCTs [15-17]. Two RCTs were conducted in China [15,16] and one in Korea [17]. All RCTs adopted a two-arm parallel group design and followed Chinese medicine (CM) theory for acupoint selection. Two of the RCTs used response rates for each intervention, and outcomes were typically divided into three categories, namely (1) recovery or marked improvement, (2) improvement and (3) ineffective [15,16], based on the physicians' assessments of change in the patients' symptoms. The other one employed the outcomes with stool frequency and CAS [17].

### Risk of bias

All three RCTs had a high risk of bias. None of the RCTs described sequence generation or blinding of the assessors, complete outcome measures and allocation concealment. Adverse events were mentioned in only one RCT [17].

### Description of individual studies

Du *et al. *[15] assessed the effectiveness of moxibustion on symptoms of postpartum constipation. A total of 160 patients were divided randomly into two groups, namely moxibustion group (*n *= 80) and glycerin enema (control) group (*n *= 80). While all patients from the moxibustion group reported improved symptoms at the end of the treatment period, only 78.75% did so in the control group (significant difference between two group, *P *< 0.01).

Li and Fang [16] tested the therapeutic effects of moxibustion at *Shenque *(CV8). A total of 60 patients were randomized into two groups, namely moxibustion group (*n *= 40) and glycerol suppositories and glycerin enema (control) group (*n *= 20). The response rate was 97.5% in the moxibustion group and 65.0% in the control group (significant difference between two group, *P *< 0.01).

Kwon and Park [17] investigated the effects of moxibustion on constipation in stroke patients. A total of 36 patients were randomized into two groups, namely moxibustion group (*n *= 17) and untreated (control) group (*n *= 19). There were significant differences in frequency of bowel movements (*P *= 0.001) and the Constipation Assessment Scale (CAS) (*P *= 0.001) between the moxibustion group and control group. The stool consistency, however, was not significantly different between the groups (*P *= 0.429).

We had originally intended to conduct a formal meta-analysis. However, statistical and clinical heterogeneity prevented us from doing so.

## Discussion

All these three RCTs on the effectiveness of moxibustion for constipation were not methodologically rigorous. These trials suggested favorable effects of moxibustion to treat constipation in postpartum women [15], healthy persons [16] and patients with CVD [17]. However, all three RCTs had a high risk of bias. Moreover, they did not blind patients or assessors, record dropouts and withdrawals, implement allocation concealment and report ethical approvals. The number, quality and sample size of these trials were too low for us to draw a definitive conclusion.

Stool frequency per week and CAS are the most convenient measurements for constipation. Only one [17]of the three RCTs employed CAS and stool frequency as outcome measures while the two [15,16]failed to use validated endpoints. Without established reliability and validity, the outcome measures are subject to bias and are not comparable among trials.

The types of constipation and the diagnostic methods used in these trials may cause concern. Two RCTs investigated the effects of moxibustion on constipation secondary to postpartum [15] and stroke [17] whereas the third RCT compared moxibustion to drugs in otherwise healthy subjects with constipation [16]. Subjects in two RCTs met the Rome II criteria [15,17] whereas the third one only described Chinese medicine diagnosis [16].

An effective placebo/sham control for acupuncture or moxibustion studies is required for future studies. If we assume that the effects of moxibustion could come from stimulating acupuncture points with heat, sham moxibustion paradigms may include treating patients on non-acupoints or preventing heat stimulation on acupoints. Two sham moxibustion devices designed to minimize heat transfer have been made available [18,19].

Limitations of the present review (and indeed systematic reviews in general) pertain to the incompleteness of the evidence. The present review posed no restrictions on the publication language and searched 12 databases. However, the distorting effects of publication bias and location bias on systematic reviews and meta-analyses may still have played a role in the present review [20-22]. Further limitations include the paucity and often suboptimal quality of the primary data. Lastly, all three RCTs were conducted on Asian populations; therefore the results are only limited to Asian populations.

Further studies should include non-Asian subjects as these three trials were conducted on Asian subjects only.

## Conclusion

Current evidence from these three randomized controlled trials is insufficient to suggest that moxibustion is an effective treatment for constipation. More rigorous studies are warranted.

## Abbreviations

CAS: Constipation Assessment Scale; CCT: controlled clinical trial; CVD: cardiovascular disorders; n.r: not reported; RCT: randomized clinical trial; GI: gastrointestinal;

## Competing interests

The authors declare that they have no competing interests.

## Authors' contributions

MSL and EE designed the study and interpreted the data. TYC and JEP searched and selected the trials, and extracted, analyzed the data. MSL drafted the manuscript and EE revised the manuscript. All authors read and approved the final version of the manuscript.

## References

[B1] Ernst E Pittler MH Wider B Boddy K The Desktop Guide to Complementary and Alternative Medicine 2006 Philadelphia: Mosby Elserviser

[B2] Tillisch K Complementary and alternative medicine for functional gastrointestinal disorders Gut 2006 55 5 593 596 10.1136/gut.2005.078089 16609129 PMC1856117

[B3] Tillisch K Complementary and alternative medicine for gastrointestinal disorders Clin Med J R Coll Physicians 2007 7 3 224 227 10.7861/clinmedicine.7-3-224 PMC4952695 17633940

[B4] Vlieger AM Blink M Tromp E Benninga MA Use of complementary and alternative medicine by pediatric patients with functional and organic gastrointestinal diseases: results from a multicenter survey Pediatrics 2008 122 2 e446 451 10.1542/peds.2008-0266 18662934

[B5] World Health Organization Western Pacific Region WHO International Standard Terminologies on Traditional Medicine in the Western Pacific Region 2007 Manila

[B6] Lin LW Fu YT Dunning T Zhang AL Ho TH Duke M Lo SK Efficacy of traditional Chinese medicine for the management of constipation: a systematic review J Altern Complement Med 2009 15 12 1335 1346 10.1089/acm.2008.0373 19958146

[B7] Zhao JP Wang YP Acupuncture and Moxibustion (Chinese Medicine Study Guide) 2008 Beijing: People's Medical Publishing House

[B8] Li GR Li QY Gemo RL Clinical Moxibustion Therapy 2008 Beijing: People's Medical Publishing House

[B9] Korean Acupuncture & Moxibustion Society Acupuncture and Moxibustion 2008 Seoul: Jibmundang

[B10] Li MK Lee T-FD Suen K-PL A review on the complementary effects of auriculotherapy in managing constipation J Altern Complement Med 2010 16 4 435 447 10.1089/acm.2009.0348 20423213

[B11] Ouyang H Chen JD Therapeutic roles of acupuncture in functional gastrointestinal disorders Aliment Pharmacol Ther 2004 20 8 831 841 10.1111/j.1365-2036.2004.02196.x 15479354

[B12] Schneider A Streitberger K Joos S Acupuncture treatment in gastrointestinal diseases: a systematic review World J Gastroenterol 2007 13 25 3417 3424 17659687 10.3748/wjg.v13.i25.3417 PMC4146776

[B13] Zhao H Liu JP Liu Z Peng W Acupuncture for chronic constipation (Protocol) Cochrane DB Syst Rev 2003 2 CD004117

[B14] Higgins JPT Altman DG Julian PTH, Green S Assessing risk of bias in included studies Cochrane Handbook for Systematic Reviews of Interventions 2008 West Sussex: Wiley-Blackwell 187 241 full_text

[B15] Du GZ Ma XD Wang CP Li SG Clinical observation of moxibustion at "tongbian Point" treatment for postpartum constipation J Hebei Med Coll Contin Educ 2008 25 5 53 55

[B16] Li YH Fang SL Moxibustion treatment for constipation of 40 cases J External Ther Tradit Chin Med 2001 10 6 12

[B17] Kwon SJ Park JS The effect of ChunChu(ST25) moxibustion on the constipation of CVA patients Clin Nurs Res 2005 11 1 179 189

[B18] Park JE Lee MS Choi SM Is it possible to blind subjects using sham moxibustion treatment? Am J Chin Med 2009 37 2 407 409 10.1142/S0192415X09006928 19507282

[B19] Zhao B Wang X Lin Z Liu R Lao L A novel sham moxibustion device: a randomized, placebo-controlled trial Complement Ther Med 2006 14 1 53 60 discussion 61 10.1016/j.ctim.2005.06.003 16473754

[B20] Dickersin K The existence of publication bias and risk factors for its occurrence JAMA 1990 263 10 1385 1389 10.1001/jama.263.10.1385 2406472

[B21] Egger M Smith GD Bias in location and selection of studies BMJ 1998 316 7124 61 66 9451274 10.1136/bmj.316.7124.61 PMC2665334

[B22] Ernst E Pittler MH Alternative therapy bias Nature 1997 385 6616 480 10.1038/385480c0 9020351

